# Factors Associated to Vaccination against Influenza among Elderly in a Large Brazilian Metropolis

**DOI:** 10.1371/journal.pone.0123840

**Published:** 2015-04-13

**Authors:** Ana Paula Sayuri Sato, José Leopoldo Ferreira Antunes, Roudom Ferreira Moura, Fabíola Bof de Andrade, Yeda Aparecida Oliveira Duarte, Maria Lúcia Lebrão

**Affiliations:** 1 Department of Epidemiology, School of Public Health, University of São Paulo, São Paulo, Brazil; 2 Center for Epidemiological Surveillance “Professor Alexandre Vranjac” of the São Paulo State, São Paulo, Brazil; 3 Center for Studies in Public Health and Aging, Rene Rachou Research Institute, Oswaldo Cruz Foundation, Belo Horizonte, Brazil; 4 Department of Medical-Surgical Nursing, School of Nursing, University of São Paulo, São Paulo, Brazil; University of Cambridge, UNITED KINGDOM

## Abstract

**Background:**

This study aimed to estimate coverage and identify factors associated to vaccination against influenza in the elderly population.

**Methods:**

The study design was cross-sectional and population based. Data was collected in 2010 by the Health, Well-Being and Aging Study. Sample consisted of 1,341 community-dwelling elderly, in São Paulo, Brazil. Association between vaccination and covariates was evaluated by means of prevalence ratios estimated by Poisson regression models.

**Results:**

Self-reported vaccination coverage was 74.2% (95% confidence interval: 71.3–76.9). Remaining physically active and having had recent interaction with health services, mainly with public units of healthcare, were the main incentives to increase vaccination coverage among the elderly; whereas lower age, living alone and absent interaction with health services were the main constraints to influenza vaccination at the community level. These covariates had already been reported to influence influenza vaccination of elders in previous years.

**Conclusion:**

Previous knowledge already available on the main constraints to influenza vaccination has not allowed to remove them. Influenza campaigns should be strengthened to increase vaccination coverage, especially in the group more reticent to vaccination. Instructing healthcare providers to recommend vaccine uptake is an important piece of this puzzle.

## Introduction

Influenza is a highly transmissible viral illness that may lead to severe complications from underlying diseases, primary viral or secondary bacterial pneumonia and death. Elderly people suffering from chronic medical conditions or immunological disorders are especially vulnerable to the disease. Persons aged 65 or more years old account for approximately 90% of all influenza-related deaths [1].

Early vaccination against influenza is considered a cost-effective strategy to prevent transmission and reduce influenza-related morbidity and mortality [1–2]. Influenza vaccination has been implemented in Brazil since 1999, without out-of-pocket expenses. Immunization campaigns have had nation-wide amplitude and relevant media involvement [3]. The first campaign of influenza vaccination for the elders in the country occurred in the city of São Paulo in 1998. More than 70% of people aged 60 or more years old were vaccinated in that year, though vaccination coverage reduced subsequently, until 2003 when the goal of vaccinating more than 70% was again accomplished. Vaccination coverage increased thereafter, and in 2008 a coverage of 80% was proposed as the new baseline for the whole country [4].

The outbreak of pandemic influenza in São Paulo in 2009 increased the importance of vaccination as a public health strategy. In 2010, vaccine formulation in Brazil encompassed pandemic and seasonal, type A and type B influenza viral strains. More than eight million individuals were vaccinated against influenza in the city of São Paulo in 2010, including children, pregnant women, healthcare providers, people with chronic diseases, adults (20–40 years of age) and elders [3].

However, the new goal of vaccination coverage among the elderly was not achieved in the city [5], and heterogeneous frequencies of population vaccinated have been reported in different Brazilian cities and regions [6–9].

Some studies have reported factors that contribute for vaccination among the elderly: older age, white race, being married, higher education, non-smoking status, being physically active, having poor physical health or a personal history of chronic conditions and being recommended by a doctor [9–14]. Complementarily, the fear of adverse effects, disbelief in vaccine effectiveness, lack of information on the theme and lack of recommendation by health professionals influence the decision of elderly people to do not uptake the influenza vaccine [15–16].

These observations reinforce the need of a continuous monitoring of vaccination coverage and factors associating with the likelihood of being vaccinated in the Brazilian context.

This study aimed at assessing the proportion of elders that were vaccinated against influenza in the city of São Paulo in 2010 and the main covariates for this outcome.

## Methods

This epidemiological, population-based study followed a cross-sectional design. Participants were enrolled in a multistage sampling design, representative of community-dwelling elderly individuals (60 or more years old) living in the city of São Paulo, Brazil. Ethical clearance was provided by the Ethics in Research Committee of the School of Public Health, University of Sao Paulo. Participation was voluntary and all participants signed a statement of informed consent.

Data came from SABE study—Saúde, Bem-estar e Envelhecimento (Health, Wellbeing and Ageing Study), a longitudinal study which began in 2000 (n = 2,143) and has been repeated every five years (2006 and 2010). The original sample observed a complex, multistage sampling design planned to allow statistical inference for the urban population aged 60 years and older. The city's census tracts were the primary survey units, and households were the secondary survey units. All individuals aged 60 or more years old living in the selected households were invited to participate.

During the follow-up study, in 2006 and 2010, two new cohorts of people aged 60 to 64 years were included, in order to maintain the original age range represented in the sample. In 2006, 1,115 participants of the initial sample remained in the study and 298 individuals aged 60 to 64 years old were included (n = 1,413). In 2010, 748 survivors from the initial sample and 241 from the 2006’s cohort remained in the study. Finally, 355 new participants aged 60 to 64 years old were included in 2010 (n = 1,344).

From the overall sample, three participants (who have not informed whether they had or had not been vaccinated in 2010) were excluded and considered sample loss, resulting a final sample of 1,341 individuals. Sample weights applied in 2000 and 2006 were reassessed to allow statistical inferences relative to the population aged 60 or more years old in the county.

All participants were interviewed in their own households. Specifically trained health professionals applied a comprehensive questionnaire on socio-demographic characteristics, behavior, quality of life, use of health services and clinical information, in addition to some physical tests. Further details pertaining to the study methodology were previously reported in other publications [17–18], and additional information (methodology, sample design and questionnaire) on the SABE study can be found at http://www.fsp.usp.br/sabe/.

The outcome variable of this study was the answer to the direct question on having or having not been vaccinated against influenza in 2010. The assessment of factors associated with this outcome considered socio-demographic characteristics (gender, age, formal education, marital status, living alone, race, employment status, relative income perception), behavior (tobacco smoking, alcohol drinking, physical activity), self-reported health status (perception of health status, number of chronic diseases, hypertension, diabetes, cardiovascular disease, fall, depression, bedridden), and use of health services (health care in the last year, type of health service and hospitalization in the last year).

Socio-demographic covariates were included in the assessment in order to identify unfair, unnecessary and avoidable inequalities in the outcome of being vaccinated. Behavioral covariates were assessed as proxies of commitment with a healthy lifestyle. Covariates on self-reported health status were assessed as indicative of the perception of being at risk, and use of health services were included to assess the hypothesis that a closer contact with health professionals would enhance the chance of being vaccinated.

Data analysis used Poisson regression, as recommended by Barros and Hirakata [19] for cross-sectional studies assessing non-rare events. Poisson regression provides direct estimates for the Prevalence Ratio (PR), which has some advantages in comparison to odds ratios in cross-sectional studies (simplicity and more familiar arithmetic involved) [19–20].

All variables were considered in multivariate analysis, but only the significant or almost significant variables remained in the final model. The fitting of the multivariate model observed a conceptual framework organizing covariates [21]. Firstly, socio-demographic variables were included and adjusted for themselves. Behavior and self-reported health status were considered to be influenced by socio-demographic characteristics and to influence the use of health services, i.e. these covariates were exclusively adjusted for socio-demographic conditions. At last, the use of health services were adjusted for themselves, socio-demographic, behavioral and self-reported health variables

Data analysis was used the Stata 12.0 2011 software (Stata Corporation, College Station, TX, USA). Specifically, the statistical analysis used Stata's survey module, which allows to incorporate aspects concerning the complex sampling design: disproportionate stratification of primary and secondary sampling units, random selection of strata and weighting. Weights were assessed as the inverse of the sampling fraction as adjusted for the corresponding distribution of population by gender and age group.

## Results

This study interviewed 1,341 individuals, representing the population of 1,338,138 elders (60 or more years old) living in the city of São Paulo, Brazil. Overall vaccination coverage was 74.2% (95% confidence interval: 71.3–76.9).

Table 1 describes the distribution of population by socio-demographic characteristics and vaccination status in 2010. Age was the only covariate that significantly associated with influenza vaccination. Participants aged 60 to 69 years old were vaccinated to a lower extent than older individuals (p = 0.01). Vaccination coverage reached almost 80% of elders aged 70 years old or more; this proportion was nearly 70% for those aged 60 to 69 years old. The remaining socio-demographic covariates were not significantly associated with vaccination status (p≥0.05).

**Table 1:** Influenza vaccination among the elderly (n = 1,341) by socio-demographic characteristics. São Paulo, Brazil, 2010.

		Vaccinated against influenza		
Socio-demographic characteristics		Yes (%)	No (%)	Unadjusted PR* (95% CI)**
	n	n = 1009	n = 332	
**Gender**				
Male	479	73.3	26.7	0.98 (0.91–1.06)
Female	862	74.9	25.1	Reference
**Age-group**				
60–69	387	70.6	29.4	0.89 (0.81–0.97)
70–79	596	78.2	21.8	0.98 (0.91–1.06)
80 or more	358	79.3	20.7	Reference
**Education**				
0–3 yrs of schooling	530	77.5	22.5	Reference
4–7 yrs of schooling	495	71.5	28.5	0.92 (0.85–0.99)
8–10 yrs of schooling	100	71.7	28.3	0.92 (0.82–1.04)
11 yrs or more of schooling	215	74.5	25.5	0.96 (0.86–1.07)
N.A. ***	1	100.0	0.0	
**Marital status**				
Currently married	663	72.5	27.5	0.96 (0.89–1.02)
Single, divorced, widowed	662	75.8	24.2	Reference
N.A. ***	16	69.6	30.4	
**Living alone**				
Yes	223	69.2	30.8	0.92 (0.84–1.01)
No	1118	75.1	24.9	Reference
**Race / skin color**				
White	779	75.8	24.2	Reference
Non-White	550	71.6	28.4	0.95 (0.89–1.00)
N.A. ***	12	100.0	0.0	
**Employment status**				
Currently working	377	71.1	28.9	0.94 (0.86–1.01)
Retired / unemployed	958	76.0	24.0	Reference
N.A. ***	6	55.4	44.6	
**Relative income perception**			
Sufficient	748	75.3	24.7	1.03 (0.96–1.11)
Insufficient	560	72.7	27.3	Reference
N.A.***	33	76.1	23.9	

Source: SABE study, 2010.

*PR—Prevalence Ratio.

**(95% CI)- 95% Confidence Interval.

***N.A.—not answered.

Table 2 shows how influenza vaccination relates to behavioral characteristics. The proportion of vaccinated was significantly higher to physically active elders, considering 150 minutes or more of mild, moderate or vigorous physical activity per week. Influenza vaccination has not resulted significantly associated with the current assessment of tobacco smoking (ever / never) and alcohol drinking (any drink during the last three months), surrogate assessment of commitment to health behaviors, respectively in the long and in the short term.

**Table 2:** Influenza vaccination among the elderly (n = 1,341) by behavioral characteristics. São Paulo, Brazil, 2010.

		Vaccinated against influenza		
Behavioral characteristics		Yes (%)	No (%)	Unadjusted PR* (95% CI)**
	n	n = 1009	n = 332	
**Alcohol intake during the last three months**				
Yes	383	72.7	25.3	0.97 (0.89–1.06)
No	957	75.0	25.0	Reference
N.A. ***	1	100.0	-	
**Tobacco smoking**				
Never smoked	706	76.1	23.9	Reference
Ever smoked	634	72.4	27.6	0.95-(0.89–1.02)
N.A. ***	1	0.0	100.0	
**Physical activity (150min/week)**				
Yes	270	83.3	16.7	1.17 (1.08–1.25)
No	1000	71.3	28.7	Reference
N.A.***	71	74.2	25.8	

Source: SABE study, 2010.

*PR—Prevalence Ratio.

**(95% CI)- 95% Confidence Interval.

***N.A.—not answered.

The association between influenza vaccination and self-reported health status was assessed in Table 3. We observed that individuals affected by one or more chronic diseases (hypertension, cardiovascular disease, diabetes mellitus, chronic obstructive pulmonary disease, arthritis, osteoporosis) were vaccinated to a significantly higher proportion than those reporting a healthier status. Bedridden elders, participants that suffered a fall during the previous year, and those affected by depression have not had significantly different vaccination coverage than their respective counterparts (p≥0.05).

**Table 3:** Influenza vaccination among the elderly (n = 1,341) by self-reported health status. São Paulo, Brazil, 2010.

Self-reported health status		Vaccinated against influenza		
		Yes (%)	No (%)	Unadjusted PR*
	n	n = 1009	n = 332	(95% CI)**
**Perception of health status**				
Good	606	73.9	26.1	Reference
Bad	689	74.0	26.0	0.99 (0.92–1.08)
N.A. ***	46	85.3	13.7	
**Number of chronic diseases**				
0	217	66.9	33.1	Reference
1	404	75.4	24.6	1.13 (0.98–1.30)
2 or more	720	74.9	24.1	1.14 (1.01–1.27)
**Hypertension**				
Yes	905	76.5	23.5	1.10 (1.01–1.19)
No	435	69.7	30.3	Reference
N.A. ***	1	100.0	0.0	
**Diabetes Mellitus**				
Yes	334	79.1	20.9	1.09 (1.02–1.16)
No	1006	72.6	27.4	Reference
N.A. ***	1	100.0	0.0	
**Cardiovascular disease**				
Yes	324	79.9	20.1	1.10 (1.03–1.18)
No	1015	72.5	27.5	Reference
N.A. ***	2	100.0	0.0	
**Falls during the last year**				
Yes	419	75.1	24.9	1.02 (0.95–1.09)
No	921	73.9	26.1	Reference
N.A. ***	1	100.0	0.0	
**Depression**				
Yes	228	70.4	29.6	0.94 (0.86–1.03)
No	1110	75.0	25.0	Reference
N.A.***	3	100.0	0.0	
**Bedridden**				
Yes	36	75.3	24.7	1.01 (0.81–1.26)
No	1305	74.2	25.8	Reference

Source: SABE study, 2010.

*PR—Prevalence Ratio.

**(95% CI)- 95% Confidence Interval.

***N.A.—not answered.

Table 4 displays the unadjusted assessment of association between vaccination and covariates on the use of health services during one year preceding the interview. Vaccination coverage ranked significantly higher for those who have had one or more episodes of healthcare during the twelve months that preceded the interview (76.3%) than for those who have not used health services. Vaccination coverage also ranked significantly higher among those that exclusively used public health services. As refers to hospitalization, no significant difference was observed between those who have and have not been hospitalized during the previous year.

**Table 4:** Influenza vaccination among the elderly (n = 1,341) by use of health services during the last year. São Paulo, Brazil, 2010.

		Vaccinated against influenza		
Use of health services		Yes (%)	No (%)	Unadjusted PR* (95% CI)**
	n	n = 1009	n = 332	
**Received health care in the last year**				
Yes	1166	76.3	23.7	1.27(1.12–1.43)
No	172	60.2	39.8	Reference
N.A. ***	3	100.0	0.0	
**Type of health service**				
Private	530	74.4	25.6	0.96 (0.88–1.03)
Public	634	77.8	22.2	Reference
N.A.***	177	61.1	38.9	
**Hospitalization in the last year**				
Yes	153	75.7	24.3	1.02 (0.92–1.13)
No	1188	74.1	25.9	Reference

Source: SABE study, 2010.

*PR—Prevalence Ratio.

**(95% CI)- 95% Confidence Interval.

***N.A.—not answered.

Table 5 synthesizes the multiple variables analysis, with outcomes obtained by the final Poisson regression model. As refers to socio-demographic factors, the assessment of adjusted associations highlighted elders who live alone and those aged from 60 to 69 years old as the groups more reticent to influenza vaccination. Maintaining physically active was also highlighted as a factor for the outcome in the adjusted assessment. Healthier elders, those that have not demanded health care during the last year, and those using private units of healthcare have been highlighted as less inclined to be vaccinated.

**Table 5:** Multiple variables analysis: Poisson regression model for influenza vaccination among the elderly (n = 1,341). São Paulo, Brazil, 2010.

	Adjusted PR*
	(95% CI)**
**Socio-demographic characteristics (1)**	
**Age**	
60–69	0.88 (0.80–0.96)
70–79	0.97 (0.91–1.05)
80 or more	Reference
**Living alone**	
Yes	0.90 (0.82–0.99)
No	Reference
**Behavior and self-reported health status (2)**	
**Physical activity (150min/week) (a)**	
Yes	1.16 (1.08–1.25)
No	Reference
**Number of chronic diseases (b)**	
0	Reference
1	1.12 (0.97–1.29)
2 or more	1.11 (0.99–1.24)
**Hypertension (c)**	
Yes	1.08 (1.00–1.18)
No	Reference
**Diabetes Mellitus (d)**	
Yes	1.08 (1.01–1.16)
No	Reference
**Cardiovascular disease (e)**	
Yes	1.08 (1.01–1.16)
No	Reference
**Use of health services (3)**	
**Received health care during the last year**	
Yes	1.46 (1.26–1.70)
No	Reference
**Type of health service**	
Private	0.93 (0.86–1.00)
Public	Reference

Source: SABE study, 2010.

*PR—Prevalence Ratio.

**(95% CI)- 95% Confidence Interval.

(1) Socio-demographic characteristics adjusted for themselves: age and living alone

(2) Covariates assessing behavior and self reported health status adjusted for socio-demographic characteristics: a) physical activity, age and living alone; b) number of chronic diseases, age and living alone; c) hypertension, age and living alone; d) diabetes mellitus, age and living alone; e) cardiovascular disease, age and living alone

(3) Covariates on use of health services adjusted for themselves, for socio-demographic characteristics and for behavior and self reported health status: received health care during the last year, type of health service, age and living alone, physical activity, number of chronic diseases.

In synthesis, results obtained in this study emphasize that remaining physically active and having had recent interaction with health services, mainly with public units of healthcare, are the main incentives to increase vaccination coverage among the elderly people; whereas lower age, living alone and absent interaction with health services are the main constraints to influenza vaccination at the community level.

## Discussion

Having assessed the coverage of influenza vaccination in elders living in the city of São Paulo and having identified factors that influenced the likelihood of being vaccinated are the most important results of this study.

Frequency of vaccination in this study (74.2%) is consistent with the coverage estimated by the local health authority (72.3%). The small difference between these estimates can be attributed to how they were assessed. The present study was based on self-reported information, in a representative sample of community-living elders; whereas the local health authority assessed the number of doses distributed and effectively administered, including elders living in institutional settings.

Vaccination coverage in Sao Paulo was lower than the goal for influenza campaigns in Brazil, which is to vaccinate more than 80% of the target population since 2008 [3]. The World Health Organization goal is to achieve a 75% coverage in high-risk groups (the elderly, children under two years of age and people with chronic conditions) by 2010 [22]. The findings of the current study underscore the fact that both the Brazilian and the international objectives have not been fulfilled in São Paulo, 2010.

Therefore, assessing covariates that effectively influence the likelihood of vaccine uptake and strengthening ongoing public health interventions are necessary to achieve optimal vaccination rates. Previous studies conducted in Brazil [6–9] and in other countries [10–11], [23] reported analogous results as refers to factors that associated with influenza vaccination. In spite of the previous knowledge on constraints and incentives to vaccination of the elderly, the same covariates remained associating significantly with the likelihood of being vaccinated in 2010.

Among the elderly, older age has been acknowledged as a predisposing factor to vaccination [11–12], [23]. Older elderly persons may present a poorer perception of health status, which would foster their acknowledgement of vulnerability to the deleterious effects of respiratory infections. Elders that lived alone were more reticent to vaccination; this result may have been influenced by the lack of informal support provided by spouses or relatives. A sedentary lifestyle has been highlighted as another constraint to influenza vaccination among the elderly [7], [13], [24–25]. Physically active individuals are more committed to healthy behaviors and may have an easier access to healthcare facilities.

The association between vaccine uptake and the self-report of a chronic condition (hypertension, diabetes mellitus, cardiovascular disease) is also an expected result. Since 2005, the local health authority has considered people affected by chronic diseases, even those younger than 60, as a target population for influenza vaccination [26]. Individuals with diabetes, hypertension and other chronic conditions are more likely to consider themselves susceptible to influenza, and therefore, have strong motivation to seek vaccination. They are also more likely to have attended healthcare units where additional advice for vaccination may have been provided [6], [8–9], [14], [23], [27–29].

The number of yearly visits to the physician is also an important factor for a continued adherence to vaccination [30]. Vaccination is encouraged by healthcare providers; complementarily, individuals concerned with their own health status may be concurrently more inclined to attend health professionals and to be vaccinated [23]. The fact that individuals attended at public units of healthcare are more likely to be vaccinated is a relevant finding of the current study, and is in line with a previous report [23].

The recommendation by a healthcare provider plays a decisive role in the decision to vaccine uptake [25], [31–34]. Some individuals feel too weak to vaccination; they fear adverse effects and are uncertain on vaccine effectiveness [8], [10], [35–37]. Others feel too strong and do not expect to contract influenza, or think that the disease may be easily overcome [31], [38]. Both types are in need of health information, and the positive influence of healthcare workers on this issue cannot be overemphasized.

Improved socioeconomic standings have been underscored as an important predictor of influenza vaccination in the international context [36–37], [39]. Especially in low-income countries, the out-of-pocket expenditure may constitute a barrier for influenza vaccination. However, this study showed that vaccine uptake was unrelated to socioeconomic status, which is also a relevant finding. In Brazil, the National Immunization Program provides free-of-charge vaccine shots for the elderly, and previous studies also reported the absence of socioeconomic inequalities in outcomes related to influenza vaccination among the elderly [2].

The main limitation of the present study is that data on vaccination was exclusively based on self-reported information, which was not checked with medical or administrative records. This strategy is not exempt of recall bias, and may have introduced interviewer bias, which may lead to analytical bias. However, previous studies appraised the validity of self-reported information on influenza vaccination and considered this measurement to be highly sensitive and moderately specific [40–41].

We also acknowledge as a study limitation the fact that this study was undertaken soon after the outbreak of pandemic influenza in 2009, which might have influenced the perception of risk and the decision to be vaccinated. A previous study forecasted a low level of vaccination in the absence of an increased perception of risk to pandemic influenza [42]. Behavioral changes would require that the risks and consequences of pandemic influenza be perceived as highly different from seasonal influenza. However, a previous study in the city of São Paulo, conducted previously to the outbreak of pandemic influenza in 2009, reported analogous results on vaccination coverage and covariates for being vaccinated [5].

Our study stressed the fact that factors previously reported to influence vaccine uptake remained associated with this outcome in 2010. Earlier knowledge already available on the main constraints to influenza vaccination has not allowed to remove them. Influenza campaigns should be strengthened to increase vaccination coverage, and health promotion initiatives, mainly the recommendation by healthcare providers to population segments more reticent to vaccine uptake is an important piece of this puzzle.
